# The Nefarious Nexus of Noncoding RNAs in Cancer

**DOI:** 10.3390/ijms19072072

**Published:** 2018-07-17

**Authors:** Eleni Anastasiadou, Alberto Faggioni, Pankaj Trivedi, Frank J. Slack

**Affiliations:** 1 HMS Initiative for RNA Medicine, Department of Pathology, Beth Israel Deaconess Medical Center, Harvard Medical School, Boston, MA 02215, USA; fslack@bidmc.harvard.edu; 2 Department of Experimental Medicine, La Sapienza University, Viale Regina Elena 324, 0161 Rome, Italy; alberto.faggioni@uniroma1.it (A.F.); pankaj.trivedi@uniroma1.it (P.T.)

**Keywords:** noncoding RNA, miRNA, lncRNA, circRNA, ncRNA network in cancer, cancer biomarkers, RNA aided cancer therapy

## Abstract

The past decade has witnessed enormous progress, and has seen the noncoding RNAs (ncRNAs) turn from the so-called dark matter RNA to critical functional molecules, influencing most physiological processes in development and disease contexts. Many ncRNAs interact with each other and are part of networks that influence the cell transcriptome and proteome and consequently the outcome of biological processes. The regulatory circuits controlled by ncRNAs have become increasingly more relevant in cancer. Further understanding of these complex network interactions and how ncRNAs are regulated, is paving the way for the identification of better therapeutic strategies in cancer.

## 1. Introduction

A few years after the proposal by Watson and Crick of the central dogma of Molecular Biology, which suggested the unidirectional flow of genetic information from DNA to coding RNA to protein, Francis Crick’s “adaptor” hypothesis, suggested the existence of a new RNA class later discovered to be transfer-RNA (tRNA) that connects messenger RNA (mRNA) and the amino acid sequences of a protein [1]. Moreover, Walter Gilbert’s “The RNA World” hypothesis that describes a world made of RNA molecules, the ribozymes, that synthesize themselves, was inspired by the enzymatic activities of an RNA molecule, a ribonuclease-P (RNP) discovered in prokaryotes—*Escherichia coli* and the ribosomal RNA that contains a self-splicing exon discovered in eukaryotes—*Tetrahymena* [2,3,4]. Many other discoveries followed, but still most scientific efforts were biased towards coding gene discovery. Furthermore, at that time there were few methodologies to detect the non-coding portion of our genome.

*Lineage defective-4* (*lin-4*) and *lethal-7* (*let-7*) were the first microRNAs (miRNA) identified, both found in *Caenorhabditis elegans* [5,6]. This was followed by a series of studies dealing with the biogenesis and function in physiological and pathological conditions of these tiny non-coding RNA oddities. Now functional non-coding RNAs (ncRNAs) have become the scientific penchants since they constitute almost 60% of the transcriptional output in humans [7,8]. As the name suggests, the ncRNAs are not transcribed into proteins but they are remarkable regulators of important molecular processes in species ranging from *Arabidopsis thaliana* and *Caenorhabditis elegans*, to humans [9]. 

The ncRNA family is generally divided into two main classes: housekeeping ncRNAs and regulatory RNAs. The housekeeping ncRNAs are involved in essential cellular functions such as ribosomal RNA (rRNA), transfer RNA (tRNA), small nucleolar RNAs (snoRNAs) and are generally expressed constitutively. Regulatory ncRNAs are involved in gene regulation and include small interfering RNAs (siRNAs), P-element-induced wimpy testes (piwi)-interacting RNAs (piRNAs), enhancer RNAs (eRNAs), miRNAs and the long-noncoding RNAs (lncRNAs), which include the class of circular RNAs (circRNAs) [10]. Their length matters when it comes to detecting and distinguishing them by RNA deep sequencing analysis so their classification is based mainly upon the number of nucleotides (nt), i.e., 20–22 nt for miRNAs and more than 200 nt for lncRNAs and the circRNAs.

Among the ncRNAs, miRNAs are the most studied, as a post-transcriptional gene regulator involved in many human phenomena and disease states. According to the last version of miRBase 21 there are a total of 28,645 hairpin precursor miRNAs that produce 35,828 mature miRNAs in 223 species [11]. One gene can be targeted by many miRNAs and one miRNA targets many genes-through RNA base-pairing and the outcome of this interaction is the post transcriptional inhibition of the target, or contrarily, can enhance the expression of a gene under specific cellular conditions and we will examine some paradigms in this review. Proteins may also influence expression of miRNAs by interfering with their biogenesis. As discussed below, the fact that miRNAs interact with circRNAs and lncRNAs, makes the scenario even more intriguing.

The discovery of RNA splicing, in 1977 by Phillip A. Sharp and Richard J. Roberts and one year earlier, of the covalently closed RNA structure of viroids, both became the springboards of discovery of circRNAs, a class of lncRNAs [12,13,14]. They were first observed by electron microscopy, 38 years ago, in the cytoplasm of eukaryotic cells [15]. Later in 1991, while Janice Nigro et al. were characterizing the tumor suppressor gene *Deleted in Colorectal Cancer* (*DCC*) in rats and in humans and in neoplastic and normal cell types, they identified novel products of splicing, named “scrambled exons” derived from a trans-splicing between exons that contain complementary sequences in distant regions of their introns [16]. Subsequently, Capel et al. in 1993 found that Sex determining region Y (SRY) c-DNA and 5′-Rapid Amplification of cDNA Ends (RACE) clones, isolated from mouse testis had an unusual circular structure and were abundant, reaching a whopping 90% of all transcripts [17]. In the same year, another group identified mis-spliced, highly stable, circRNAs of the human proto-oncogene transcription factor *ETS-1* [18]. A few years later, Zaphiropoulos et al., hypothesized that circRNAs may be formed also through an exon skipping mechanism [19]. In other words, the crossover point of the covalently closed loop brings together alternate 5′ donor splice sites with the 3′ splice acceptor sites of non-sequential exons, forming circRNAs. Their length may vary from more than 200 nt to less than 100 nt. Only in the last few years have we started to understand their biogenesis. The main reason behind this “neglect” being that they were considered cytoplasmic byproducts of splicing. Additionally, their identification was missed due to molecular techniques of those days, such as RNA sequencing analysis, which enriched only poly(A) RNA samples and excluded the “back spliced” circRNAs because they don’t have poly(A) tail. More recently, thousands of circRNAs in different species, *Drosophila Melanogaster, Caenorabditis elegans*, mouse and humans have been identified but little is known about their biogenesis, function and biological significance [20,21,22].

Overall, the previously mentioned classes of ncRNAs have critical functions in post-transcriptional oncogene and tumor suppressor regulation. Also, they may interact or compete with one another. These interactions are part of the competitive endogenous RNAs (ceRNAs) hypothesis, that includes pseudogenes which lack coding potential and can include lncRNAs, circRNA that compete for the same group of miRNA responsive elements (MRE) in the transcriptome and may have implications in tumorigenesis [23,24,25].

The review will start with a comparison between miRNA, circRNA and lncRNA biogenesis. Subsequently, we will focus on the function and regulation of the three classes of ncRNAs in cancer. In addition, recent insights into how interactions between the various classes of ncRNAs drive or suppress oncogenesis will be examined. An exploration of these topics is timely, given the flood of new publications analyzing the role of miRNAs, lncRNAs, and circRNAs in cancer [26,27]. Since, novel RNA based therapeutics have started to become an intergral part of clinical applications, it becomes imperative to understand the complexity of ncRNA networks to ensure that such technologies are applied to cancer treatment [28].

## 2. Comparison of the Biogenesis of Noncoding RNAs

**Figure 1:**
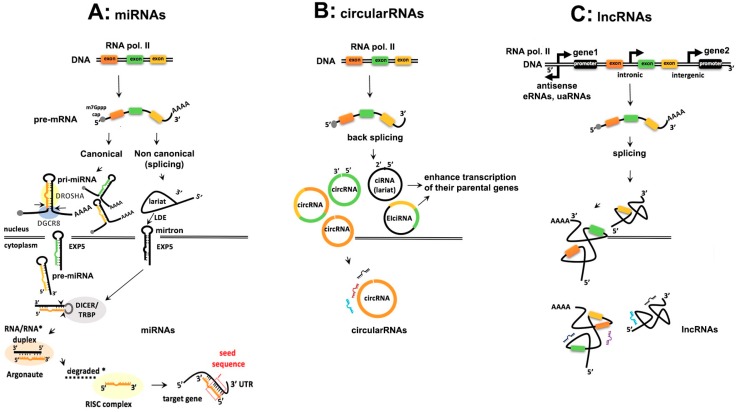
An overview of biogenesis of (**A**): miRNAs, (**B**): circular RNAs and (**C**): lncRNAs. NcRNAs are first generated in the nucleus, transcribed by polymerase II giving rise to a long, sometimes hundreds of kilobases 5′ capped and 3′ polyadenylated transcripts. (**A**): Canonical miRNA: DGCR8 and Drosha bind and cleave the basal stem of pri-miRNAs to liberate the stem-loop pre-miRNA that enters the cytoplasm by Exportin 5 (EXP5) where the pre-miRNA loop is cleaved by the RNase III Dicer and HIV-1 TAR RNA binding protein (TRBP) to form a mature RNA duplex. One of the two strands, the guide strand, is selected by one of the four Argonaute (AGO) proteins and loaded together with the passenger strand in the RNA induced silencing complex (RISC). The passenger strand is degraded while the guide strand will become the mature miRNA. Non-canonical miRNAs: mirtrons: reside in introns at the exon junction site of the pre-mRNA, are spliceosome-dependent and have their own processing mechanism, which is independent from microprocessor complex. Mirtrons form lariats which are debranched under the action of lariat debranching enzymes (LDE) to fold into the pre-miRNA hairpin and proceed through the canonical miRNA pathway maturation. (**B**): Circular RNAs: The canonical spliceosomal machinery produces 3′-5′ backspliced circular pre-mRNA transcripts that are composed of exons (exonic circRNAs), or 2′-5′ lariats (intronic RNAs, ciRNAs) and exon-intron circRNAs (EIciRNAs). CiRNAs and ElciRNAs remain in the nucleus in contrast to the circRNAs, which are transported in the cytoplasm. (**C**): LncRNAs: Based on their location in the genome, lncRNAs may be long intergenic (lincRNAs), between two adjacent genes or intronic or within the intron of a protein-coding gene. Antisense lncRNAs as uaRNAs or eRNAs are transcribed in an opposite direction from the host gene. Intergenic and intronic lncRNAs are spliced from the pre-mRNA, through the spliceosomal machinery and can be polyadenylated or not. One of the functions of lncRNAs is to decoy miRNAs.

The exploration of different steps involved in ncRNAs biogenesis is necessary because the deregulation of the biogenesis may have implications in oncogenic pathways. Here, we compare the biogenesis of miRNAs (Figure 1A), circular RNAs (Figure 1B) and lncRNAs (Figure 1C), highlighting the common aspects, differences as well as some exceptions of noncanonical ncRNAs, that may follow different pathways during their final maturation. A broader understanding of biogenetic mechanisms will provide also the right tools to understand ncRNA dependent tumorigenesis better and consequently devise better therapeutic tools.

### 2.1. Similarities and Differences in Biogenesis of ncRNAs

#### 2.1.1. Transcription, Capping and Adenylation

Similarities: Generally, ncRNAs are generated in the nucleus, transcribed by polymerase II (Pol II) giving rise to a long, usually hundreds of kilobases 5′ capped and 3′ polyadenylated transcript, the precursor mRNA (pre-mRNA) (Figure 1A–C). But within the lncRNAs the predominant form may be monoexonic and nonpolyadenylated, particularly in the case of eRNAs and the promoter-associated upstream antisense lncRNAs (uaRNAs) [29]. The ncRNA genes are interspersed throughout the genome, and can be intergenic, or intronic.

miRNAs: Intergenic miRNAs (i.e., *lin-4* and *let-7*) are independently transcribed, in contrast with the intronic miRNAs (i.e., miR-26) that reside in their host gene and are transcribed from the same promoter as the host gene itself [30,31]. Furthermore, intronic miRNAs need RNA splicing components to be spliced out from the transcript of their host gene and become mature miRNA. Whether miRNAs are intergenic or intronic, they can be co-transcribed from a single transcription unit, named polycistron. A known example is the polycistronic miR-17-92 cluster, which contains six precursor miRNAs within about 1 kb on chromosome 13 [32].

CircRNAs: They are divided in intronic (ciRNAs), exonic circRNAs and exonic-intronic, (ElciRNAs) in which exons are circularized with introns (Figure 1B). [33,34,35]. It is not yet completely clear whether circRNAs are generated co-transcriptionally (while the introns are being spliced out from the pre-mRNA) or post-transcriptionally [36]. But experimental evidence in the yeast *Saccharomyces cerevisiae* and in the nuclear extract of the human cell line HeLa suggest that circRNAs are generated by an inverse splicing mechanism of the introns mediated by the spliceosome, giving rise to 3′-5′ back splice circRNA [37]. The latter may be also formed through an exon skipping mechanism as it has been demonstrated in cytochrome P-450 2C18 transcript, which is expressed in the human epidermis and in the *Androgen-binding protein* (*ABP*) gene in rat testis [19]. ElciRNAs are also derived from a backsplice mechanism driven by intron-pairing. The ciRNAs instead, have a 2′-5′ junction and form a lariat RNA [38]. All three classes of circRNAs are derived from the non-polyadenylated fraction of RNA (poly(A)^−^). 

LncRNAs: Based on their location in the genome, lncRNAs are long intergenic (lincRNAs), between two adjacent genes or intronic, within the intron of a protein-coding gene (Figure 1C). Generally, a very common characteristic of lncRNAs is the bidirectional or divergent transcription generated by Pol II, from shared protein-coding gene promoters [39]. Divergent transcription of lncRNAs is enhanced by the chromatin remodeling complex, SWItch/Sucrose Non-Fermentable (SWI/SNF) and is inhibited by chromatin assembly factor-1 (CAF1) (further details of lncRNA biogenesis are reviewed by Quinn et al. [40]). It is also worthy to mention that DICER1, a protein that aids miRNA biogenesis, along with the transcription factor c-myc, induces lncRNA transcription from divergently transcribed protein-coding genes [41]. Contrarily, four chromatin remodeling factors inhibit lncRNA transcription and this inhibition consequently regulates mRNA gene expression [42]. 

Exceptions within the group of miRNAs: Recently published reports suggest that some intronic miRNAs, like miR-93, may also have their own promoters [43]. Contrarily, intergenic or exonic miRNAs, like let-7c, are transcribed from promoters that are in the opposite direction of the gene. Not only Pol II but also RNA polymerase III (Pol III) can transcribe almost 20% of the human miRNAs because of the presence of Arthrobacter luteus restriction endonuclease (Alu) transposable elements upstream of miRNA promoters. A genomic analysis of 42 miRNAs, in almost 100 kilobase pairs (kbp) long fragment in the human chromosome 19 miRNA cluster (C19MC), had Alu sequences upstream of their promoters and were transcribed by Pol III [44].

#### 2.1.2. Processing of ncRNAs

Similarities: The pre-mRNA transcript is processed by the spliceosome. While for circRNAs and lncRNAs, this is the main processing mechanism, it is not the case for every miRNA. There are non-canonical miRNAs, the mirtrons [45], which are found in introns at the exon junction site of the pre-mRNA, and are spliceosome-dependent. In this section, we will also describe some exemptions to the canonical processing of lncRNAs.

miRNAs: In the nucleus, the microprocessor complex of the RNA-binding protein Di George Syndrome Critical Region Gene 8 (DGCR8) together with the RNase III enzyme Drosha, binds and cleaves the basal stem of pri-miRNAs to liberate the stem-loop precursor miRNA (pre-miRNA) of about 65 nucleotides (nt). Drosha contains a tandem of RNase III domains (RIIIDs) and a double strand (ds)-RNA binding domain and each domain has a specialized action to cleave accurately at the 11th bp of the hairpin away from the basal single strand junction of the 5′ strand and 22 bp from the apical junction. Also, the 3′ strand side of the hairpin is cleaved. These Drosha cleavages create a precise 2nt overhang from the 3′ side of the stem loop (Figure 1A). 

Pri-miRNA, after being processed into a pre-miRNA is exported from the nucleus into the cytoplasm by Exportin 5 (EXP5) (encoded from *XPO5* gene), supported by a Ran-GTP-dependent mechanism. Furthermore, EXP5 protects pre-miRNA from nucleolytic attack in the nucleus and enables the interaction between the stem loop and the inner surface. Once in the cytoplasm, the Pre-miRNA loop is cleaved by the RNase III Dicer and HIV-1 TAR RNA binding protein (TRBP) to form a mature RNA duplex of almost 22 nucleotides (nt) long with a 3′-end having a two nucleotides overhang. The 5′ strand of the RNA duplex or rarely the 3′ strand which contains thermodynamically unstable nucleotides, will be selected as the guide strand, which will become the mature miRNA [46]. This thermodynamic instability can facilitate the unwinding of the duplex by helicases, such as Gemin3 or RCK/p54 [47,48]. The guide strand is selected by one of the four Argonaute (AGO) proteins due to the presence of Uracil (U) at nucleotide position 1 or Adenosine (A) at the 5′ end of the RNA duplex and loaded together with the passenger strand in the RNA induced silencing complex (RISC) [49,50]. Subsequently, the passenger strand is cleaved and generally degraded, but may also be maintained or activated [51]. The 22 nt long mature miRNA contains the “seed sequence” (2 to 7 nucleotides) at the 5′ end for target recognition. Likewise, for the target recognition, complementarity at position 8 and conserved adenosines flanking the seed complementary sites in mRNAs are critical [52,53]. The interaction between the seed sequence and the 3′UTR of the target mRNA impair the translation and stability of the mRNA transcripts and this interaction occurs not only in the cytoplasm but occasionally also in the nucleus [54,55]. 

Exceptions in processing of miRNAs: In a Drosha-deleted human cell line, the canonical miRNA production was completely abolished except for a few non-canonical miRNAs (mirtrons) that were Drosha-independent and located in the introns of the mRNA encoding host genes [56]. This indicates that Drosha processing of the canonical miRNAs is indispensable for their maturation. The pre-miR-451 is the only exception so far of a pre-miRNA that escapes Dicer processing, by binding directly to Ago2 [57]. miRNAs not associated with RISC are detected in the cytoplasm as miRNA-mRNA duplexes not bound to Agos. Another exception is that also the 3′-end of a mature miRNA contributes to target recognition [58,59,60]. 

CircRNAs: A chromatin-bound RNA nascent sequencing dataset showed that circRNA and canonical splicing pre-mRNA antagonize each other and this event is tissue specific and conserved in *Drosophila* and humans [20]. The canonical spliceosomal machinery produces backspliced pre-mRNA using cis regulating mechanisms such as long bordering intronic complementary sequences, the Alu repeats in humans [61,62]. An example of a human gene locus that contains Alu repeats is the Homeodomain Interacting Protein Kinase 3 (HIPK3) locus circularization. Trans regulating factors, are also involved, like the splicing factor Muscleblind (MBL), that promotes circRNA biogenesis in *Drosophila* and it is conserved also in mice and in humans [20]. Interestingly, MBL not only binds to its own exon but also regulates its own circularization. The circularization efficiency seems to be inversely correlated with the exon length and the intronic complementary sequences which promote circularization [63].

Importantly, ciRNAs processing depends on consensus RNA sequences next to the 5′ splice site and branch point that probably makes them resistant to debranching enzymes [64]. They are localized in the nucleus and enhance transcription of their parental genes [38]. ElciRNAs are also localized in the nucleus and interact with small nuclear RNA (snRNA), like U1, that both interact with Pol II at the promoters of parental genes and activate their transcription [33]. CircRNAs are transported in the cytoplasm and are very stable because they lack 3′ and 5′ ends and consequently are protected from any exonuclease activity [35]. 

LncRNAs: The processing of lncRNAs is similar to the pre-mRNAs processing but there are some exceptions: Upon Pol II transcription, some of the newly formed lncRNAs, like MALAT1 (metastasis- associated lung adenocarcinoma transcript 1) and NEAT (nuclear enriched abundant transcript 1), undergo a particular type of the tRNA-like structure processing at their 3′-ends. The tRNA-like structure at the 3′-end is cleaved by the ribonuclease P (RNaseP) and generates tRNA-like smallRNA products and a stable 3’ triple helix containing lncRNAs. The tRNA-like smallRNAs cleaved from NEAT are unstable whereas those derived from MALAT1 are stable and cytoplasmic [65]. Another exception is the formation of hybrid molecules such as lnc-pri-miRNA and sno-lncRNAs. It has been estimated that 17.5% of miRNAs are located in lncRNAs. Examples of these hybrids are: lnc-pri-miR-21 and lnc-pri-miR-122 [66]. The microprocessor complex, that generates the pre-miRNAs, cleaves the lnc-pri-miRNA molecule and gives rise to an unstable non polyA lncRNA and a pre-miRNA so the cell can express high levels of miRNA without competing with the transcription of the host gene, the polyA lncRNA [66]. The sno-RNA related lncRNAs (sno-lncRNAs) are generated from introns between two sno-RNAs [67]. After splicing sno-lncRNAs can be produced by either box C/D or box H/ACA snoRNAs and contain snoRNA flanking sequences but deprived of a 3′ poly(A) tail and 5′ cap [68]. Their functional role in cancer is discussed in Section 3.3, dealing with lncRNAs in cancer.

## 3. Functions of ncRNAs in Cancer

### 3.1. Deregulation of miRNA Expression in Cancer

#### 3.1.1. miRNA Signatures in Cancer

Many types of cancers are characterized by a specific miRNA signature [69,70,71]. For example, miR-155/BIC precursor was found to be highly expressed in pediatric Burkitt lymphoma (BL), while reduced levels of miR-143 and miR-145 were found in the adenomatous and cancer stages of colorectal neoplasia [72,73]. Two different miRNA signatures characterize Chronic Lymphocytic Leukemia (CLL) samples and were associated with the presence or absence of mutations. In addition, significant differences were observed in miRNA expression in CLL versus normal CD5^+^ B cells [74]. Furthermore, increased levels of oncomiRs miR-21, miR-155 and the miR-17-92 were found in several lymphomas and leukemia and conversely the expression of tumor suppressors *let-7* and miR-34 is frequently decreased [75,76,77,78,79,80,81,82]. Profiling of 217 mammalian miRNAs differentially expressed in 334 human samples across different types of cancers through a bead-based flow cytometric method and validated by Northern blots, showed that miRNAs not merely characterize a type of cancer, but their expression pattern reflects the developmental history of human cancers [70]. Furthermore, miRNA expression seems higher in normal rather than in tumor tissues. This suggests that the impaired cellular differentiation associates with a decrease in miRNA expression. miRNA signature in multiple human cancers reveal the possibility to classify many tumor types and their differentiation stage based on a specific miRNA expression and their potential use in cancer diagnosis and eventually also in cancer treatment. 

One of the known miRNA signatures is the high expression of miR-155 and miR-21 in hematological malignancies and especially in lymphomagenesis. In the Eμ-miR155 transgenic mice, under the control of a VH promoter-Ig heavy chain Eμ enhancer, miR-155 activated the pre-B stage of differentiation [83]. When its expression was induced, the mice developed leukemia and high-grade lymphoma. In another study, two mice lymphoma models, miR-21 and miR-155 causes recombination- locus of X-over P1 (Cre-loxP) tetracycline-controlled knock-in mouse, were developed [76]. These mice upon induction of miR-21 and miR-155, after three months rearing in the absence of doxycycline food, developed acute lymphadenopathy and splenomegaly. The same mice when fed back with doxycycline food, completely recovered from all disease symptoms and the lymphadenopathy regressed. Anti-miR-21 and anti-miR-155 were placed in nanoparticles composed of (polylactic-co-glycolicacid) (PLGA) and the Peptide nucleic acid (PNA) with a low pH-induced transmembrane structure (pHLIP) and were successfully delivered to the mice and reduced the tumor growth. This study opened a new field of RNA-based therapeutic treatment of lymphoma. In a recent study, miR-21 encapsulated in tumor-penetrating nanocomplexes (TPN) was successfully delivered to the tumor site for pancreatic ductal adenocarcinoma (PDAC), on patient-derived-xenograft (PDX) samples and patient-derived-organoids (PDO) [84]. These findings demonstrate a new paradigm that paves the way in the use of antimiRs as anticancer drugs.

#### 3.1.2. Cellular miRNAs Interact with Viral Proteins

miRNAs interact with viral proteins in diverse types of virally associated cancers. Interestingly, about 15–20% of cancers have infectious etiology. Indeed, a broad miRNA profiling of different viral serotypes suggested that Human Papilloma virus (HPV)-16 and -18 can both upregulate certain common miRNAs like miR-25, -92a, -93, -106b, both in tumor tissues as well as in, in vitro infected foreskin derived and vaginal keratinocytes [85]. Furthermore, miR-122 is highly expressed in hepatocytes, targets the 5′ end of the genomic RNA of Hepatitis C Virus (HCV) and induce the stability and proliferation of HCV [86]. Many viruses also have evolved viral orthologues of oncogenic cellular miRNA with identical seed sequences. Oncogenic miR-155 is a case in point, viral orthologues of which, are found in genomes of KSHV and Marek’s disease virus (MDV) [87]. Epstein-Barr virus (EBV) is associated with a wide variety of cancers and contributes to cellular transformation in two ways, either by employing its own miRNA (V-miRNAs) or by altering cellular miRNA expression through virally encoded latent growth transformation proteins, EBNA2 and LMP1 [88,89,90]. The virally encoded nuclear protein, EBNA2, essential for EBV’s transforming potential, was shown to simultaneously increase miR-21, a noted oncomiR and downregulate miR-146a, which is considered a tumor suppressive miRNA [91,92]. LMP1 on the other hand, induces miR-155 expression [93]. The same viral protein, when expressed at high level increases expression of miR-29b to downregulate TCL1 expression [94]. miR-21 is consistently increased by EBV across several different types of tumors like BL and plasma cell derived malignancies [95,96]. We have recently shown that EBNA2 can increase immune checkpoint protein PD-L1 by downregulating miR-34a [28]. Cellular miRNA alteration can also be used as a biomarker of EBV. For example, the expression of specific miRNAs was either increased or decreased in the context of EBV’s presence in DLBCLs [97]. Overall, virus interaction with host cell miRNA indicate another level of viral manipulation that helps the propagation and survival of the virus and the knowledge of this interaction has implications in virally associated tumor therapies. 

#### 3.1.3. Defective Biogenesis of miRNAs

Deregulated expression of miRNAs in different cancer types may also reflect a defective miRNA biogenesis mechanism and maturation. Dicer knock-out mouse embryonic cells failed to differentiate and low levels of Dicer and Drosha expression are associated with advanced tumor stage of ovarian cancer and poor response to chemotherapy [98,99]. Contrarily, overexpression of Dicer was detected in prostate and lung cancer [100,101]. Likewise, high expression of Drosha was found in cervical squamous cell carcinoma (SCC) which, consequently altered the miRNA profile, and the cell proliferation, invasion and metastasis were enhanced [102]. A whole exome sequencing of 44 Wilms tumors, identified missense mutations in the RNase IIIB domains of Dicer1 and Drosha [103]. These mutations impaired the expression of *let-7* family as well as other tumor suppressor miRNAs, which derived from the 5′ arm of the miRNA hairpins and this finding could explain one of the main mechanisms that induce the formation of Wilms tumors. Additionally, the role of Exportin 5 (XPO5) in tumor progression has been under investigation. Decreased levels of XPO5 protein expression has been linked with a worst prognosis of renal and esophageal tumors, whereas a better prognosis has been seen in multiple myeloma, liver cancer and in non-small-cell lung cancer [104,105]. Recently, the role of XPO5 has been investigated in liver cancer [106]. Phosphorylation of XPO5 by ERK inhibits nuclear export of pre-miR-122 in the cytoplasm. Therefore, maturation of miR-122 is impaired and cannot target septin-9 (SEPT9) oncogene. Expression of this oncogene was found increased and caused Taxol resistance, a drug used in liver cancer therapy. The discovery of ERK-XPO5 pathway implies a novel therapeutic strategy towards XPO5 activation. Besides the evidence that miRNA biogenesis perturbation leads to tumorigenesis, a plethora of other mechanisms are involved as well. Importantly, it has been shown that 98 of 186 miRNA genes are found in fragile sites and in cancer associated genomic regions [107].

### 3.2. circRNAs in Cancer

Thousands of circRNAs, 2000 in human, 1900 in mouse and 700 in Caenorhabditis elegans, are detected and conserved in different tissues and developmental stages [108]. Furthermore, 25 thousand circRNAs were identified in human fibroblasts, by high-throughput sequencing analysis of ribosome-depleted and exonuclease treated RNA libraries [109]. Interestingly, they are very stable in the intracellular milieu exhibiting a half-life of more than 48 h, comparing to that of mRNAs of only 10 h or less and are also resistant to RNA exonucleases [110,111]. 

Because of the broad expression of circRNAs in various tissues and developmental stages in diverse species, a computational analysis of circRNAs, miRNAs and mRNAs interactions has been performed in different diseases, that brought to the development of Circ2Traits database [112]. This database comprises 1953 predicted human circRNAs datasets, the miR2disease dataset and human miRNAs associated with 174 different human diseases [113]. The authors of Circ2Traits database not only identified 49 circRNAs, containing one or more single-nucleotide polymorphism (SNP) in their Ago-binding sites, but they have also included the genome-wide association study (GWAS) to create a link between circRNAs associated with disease related SNPs and have predicted the circRNA interaction with disease associated miRNAs. From their study and Circ2Traits database, a circRNAs association with gastric and prostate cancer and with other diseases was unraveled. 

Another algorithm, circRNADb, includes 32,914 human exonic circRNAs collected from the literature and gives genomic information about circRNAs as well as internal ribosome entry site (IRES), open reading frame (ORF) and references [114]. CircBase, provides scripts to identify circRNAs in sequencing data [115]. The development of such algorithms reveals an increasing interest to explore interacting networks of circRNAs with miRNAs in disease and particularly in cancer. 

A ribosomal-depleted RNA sequencing analysis identified 27,000 circRNAs in six human normal tissues and seven human cancers tissues [116]. The most abundant circRNA identified in these tumors was circHIPK3 produced from exon 2 of the *HIPK3* gene, that can bind and inhibit nine tumor suppressor miRNAs, among them miR-124. When circHIPK3 was overexpressed in HEK-293T cells, the growth inhibitory effect of miR-124 was alleviated. The above findings indicate that circRNAs act as oncogenes, by sequestering tumor suppressor miRNAs. 

A computational analysis of ribo-minus RNA extracted from human umbilical venous endothelial cells (HUVEC) under hypoxic conditions, identified endothelial circRNAs [117]. One of these circRNAs is cZNF292. When this circRNA was silenced, the tube formation and spheroid sprouting were reduced in HUVEC cells, indicating that cZNF292 has proangiogenic activities in vitro. Moreover, no sponging effect for any miRNA was reported by high-throughput sequencing of RNA isolated by cross-linking and immunoprecipitation (HITS-CLIP). This is the first study, which links a circRNA with one of the hallmarks of tumorigenesis, namely hypoxia. 

### 3.3. lncRNA in Cancer

The expression of lncRNAs seems to be more specific than mRNAs in different cell types, tissues in developmental stages as well as in diseases including cancer [118,119,120,121]. As it was previously mentioned for miRNAs, also lncRNAs may act either as tumor-suppressors or promote carcinogenesis [122]. The lncRNAs are localized in the nucleus and in cytoplasm. They act in trans or in cis with transcription factors, miRNAs or other ncRNAs (sno-RNAs) and chromatin DNA. For example, enhancer-associated lncRNAs (eRNAs) regulate in trans other neighboring genes, in the nucleus, during their transcription or post transcriptionally. One known example is the regulation of HOX genes (*HOTAIR* and *HOXA*), a group of genes which controls the body orientation, that is, head-to-tail axis of animals, by the antisense RNA, HOXA transcript at the distal tip, (HOTTIP), which in turn is transcribed by the 5′ end of the *HOXA* gene. The lincRNA, HOTTIP forms a chromosomal loop, which brings together HOXA distant genes and activates their transcription [123]. This lincRNA is also known to be highly expressed across different types of cancers and associated with distal metastasis, high tumor stage and therefore may serve as a novel biomarker of poor prognosis [124]. 

**Figure 2:**
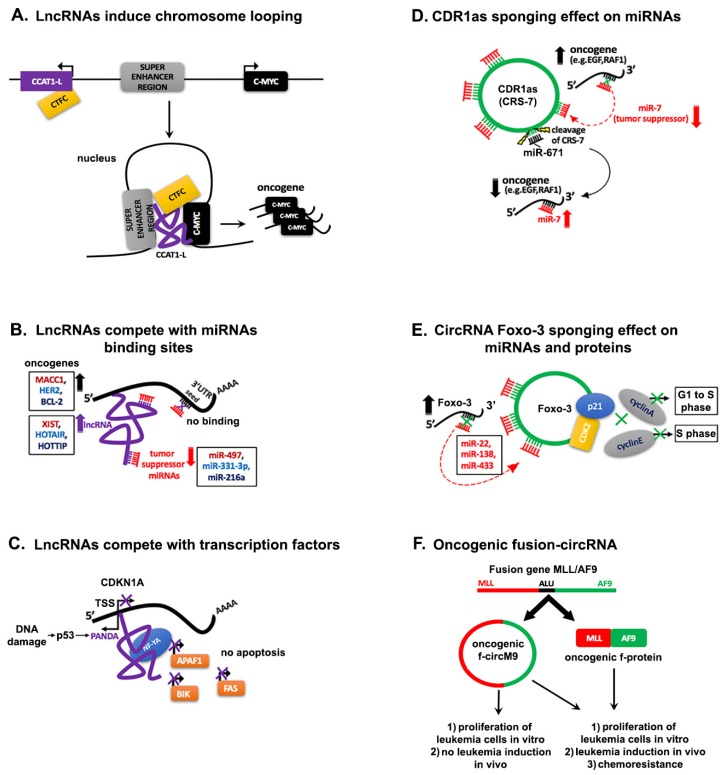
Interactions of ncRNAs in cancer. (**A**): LncRNA, CCAT1-L, interacts with the chromatin binding factor, CTCF. CCAT1-L is transcribed upstream of the oncogene *MYC* enhancer region, promotes chromosome looping that brings into proximity the super enhancer to the *MYC* promoter and induces enhanced transcription of *MYC* in colorectal cancer. (**B**): LncRNAs (XIST, HOTAIR, HOTTIP) compete with the binding of miRNA target genes. Each color corresponds to a lncRNA-oncogene-miRNA interaction. Tumor suppressor miRNAs cannot bind to their target oncogenes because they are sequestered by lncRNAs. (**C**): PANDA antisense transcription is induced by p53, counteracts CDKN1 inhibitory effect on the cell cycle and decoys the transcription factor NF-YA. The latter cannot bind anymore to the promoter of apoptotic activators such as APAF1, BIK, FAS. (TSS: transcriptional start site). (**D**): CDR1 decoys the tumor suppressor miR-7, which cannot bind to its oncogene targets. miR-671 cleaves CDR1 and alleviates miR-7 decoying. The latter is now able to suppress its targets. (**E**): Circ-Foxo3 forms a ternary complex with two cell cycle regulatory proteins, p21 and CDK2 to block the cell cycle progression. Foxo3 and circ-Foxo3 in a breast cancer cell line decreased cell proliferation and induced extensive cell death due to apoptosis in tumors formed by the same cell line in nude mice. (**F**): Chromosomal translocations of the oncogenes *MLL/AF9* in AML produce the fusion of the due oncogenes to form fusion (f)-circM9 and a fusion protein. Both, f-circM9 together with the f-protein induced leukemia in vivo and resistance to chemotherapy.

A cis acting lncRNA in the nucleus is CCAT1-L (Colorectal Cancer Associated Transcript 1-the long isoform). It is transcribed upstream of the oncogene *MYC* enhancer region and promotes chromosome looping that brings into proximity the super enhancer to the *MYC* promoter and induces enhanced transcription of *MYC* [125]. Furthermore, CCAT1-L specifically interacts with the chromatin CCCTC-binding factor (CTCF) to maintain and promote the chromatin interaction between the *MYC* promoter and its enhancers in CRC cell lines (Figure 2A). 

LncRNAs also deregulate the expression of transcriptional regulators through interaction with the Polycomb Repressive complex 1 and -2 (PRC1,-2) which modifies chromatin histones by promoting histone H3 lysine 27 trimethylation (H3K27me3) [126,127]. An example is the highly expressed lincRNA Up-regulated in bladder cancer 1 (linc-UBC1), which is associated with lymph node metastasis and poor prognosis in bladder cancer (BC) patients. Linc-UBC1 resides in the nucleus where it interacts with two proteins of the PRC2 complex, Enhancer of Zeste Homolog 2 (EZH2) and Suppressor of Zeste 12 Homolog (SUZ12). It suppresses in trans, the PRC2 target genes *BMP2, KLF4* and *HOXA5* leading to increased proliferation of BC human cell lines [128]. Like linc-UBC1, lincRNA HOTAIR also interacts with PRC2 components SUZ12 and EZH2 to promote tumor progression in breast cancer, gastrointestinal cancer and hepatocellular carcinoma [129,130,131]. 

Another lncRNA that works as molecular scaffold in the nucleus, bringing together regulatory proteins, is X-inactive specific transcript (XIST). It is required for the transcriptional silencing of one X-chromosome in female mammals, but its high expression also promotes invasion and cell proliferation in human glioblastoma stem cells, gastric cancer (GC) tissues and human GC cell lines [132,133,134]. Recently, a box H/ACA small nucleolar RNA (snoRNA)-ended lncRNA SLERT (snoRNA-ended lncRNA enhances pre-ribosomal RNA transcription) has been identified [135]. The presence of sno-RNAs at both ends of SLERT is necessary for the biogenesis and translocation for SLERT to the nucleolus, where it controls rRNA transcription by Pol I and promotes the pre-rRNA transcription through interaction with DDX21, a DEAD-box RNA helicase involved in the ribosome biogenesis. SLERT inhibits the inhibitory effect of DDX1 on Pol I, leading to enhanced transcription of rRNA, which is associated with uncontrolled transformed mammalian cell proliferation [136]. Deletion of SLERT impairs pre-rRNA transcription and rRNA production, leading to decreased tumorigenesis [135]. Among the nuclear lncRNAs, MALAT1 (also known as NEAT-2) is evolutionary highly conserved in mammals. It regulates gene expression and alternative splicing and has been linked to lung cancer metastasis and to other types of human cancers [137]. As mentioned before, there are lncRNAs, which reside in the cytoplasm where they exert their regulatory roles. One of these cytoplasmic lncRNAs is the nuclear factor kappa-light-chain-enhancer of activated B cells (NF-κB) Interacting LncRNA (NKILA). It binds to NF-κB/IκB complex and inhibits NF-κB activation, known to be involved in development and progression of human cancer. A negative feedback loop mechanism explains the initial induction of NKILA by NF-κB followed by NKILA inhibition of NF-κB. Therefore, NKILA acts as a tumor suppressor against breast cancer progression and metastasis, tongue squamous cell carcinoma cells and in non-small cell lung cancer (NSCLC) [138,139,140]. 

As expected after the discovery of the first lncRNAs in cancer like H19, MALAT1, PCA3, the list of lncRNAs involved in tumor progression and metastasis is growing and their role as prognostic biomarkers and as prominent therapeutic targets in tumor patients is becoming increasingly more evident [141,142,143].

## 4. Interaction Networks of ncRNAs in Cancer

### 4.1. Regulation through Competitive Interactions

#### 4.1.1. Competing lncRNAs

The function of lncRNAs as ceRNAs for miRNAs, transcription factors, RNA-binding proteins (RBPs), or DNAs and the fact that lncRNAs can be transcribed from pseudogenes, is a subject of intense ongoing research by several groups and these functions are important in metastasis and in tumor progression [144,145]. The exemplary interaction between a lncRNA and a miRNA in cancer is provided by the known lncRNA, XIST. As mentioned earlier, XIST is highly expressed in GC tissues compared with the normal tissues. Upon knockdown of XIST by antisense oligos in human GC cell lines, proliferation and apoptosis were also decreased. The mechanism behind the effect of XIST on cell growth and invasion in GC, involves sequestration of the tumor suppressor miR-497, which targets the oncogene *Metastasis Associated in Colon Cancer 1* (*MACC1*) (Figure 2B) [134,146]. LncRNA cancer susceptibility candidate 2 (CASC2) sequesters and suppresses onco-miR-21 responsible for the malignant progression of human gliomas and on the other hand, miR-21 targets CASC2 [147]. In gastric cancer, HOTAIR is highly expressed, and de-represses the expression of HER2 through competition for miR-331-3p binding in Ago2-containing ribonucleoprotein complex (Figure 2A) [148]. Another lncRNA, Loc285194, regulated by p53, binds and inhibits miR-211 in RISC complex in colon carcinoma cell lines [149]. LncRNA growth arrest-specific transcript 5 (GAS5) promoted proliferation, metastasis and inhibited apoptosis by regulation of miR-301a in esophageal cancer (EC) [150]. It has been recently reported that HOTTIP functions as an oncogene in small cell lung cancer (SCLC) by sponging the tumor suppressor miR-216a and consequently increases the expression of *BCL-2*, a target of miR-216a. By regulating *BCL-2* expression, HOTTIP enhanced chemoresistance of SCLC [151]. However, lncRNAs may not only sequester miRNAs but also transcription factors, as it is described in Figure 2B [152]. This is the case of the P21 associated ncRNA DNA damage activated (PANDA) lncRNA, which is transcribed antisense of the transcription start site of the cell cycle gene *CDKN1A*. Upon DNA damage by doxorubicin treatment of human fetal ling fibroblasts, PANDA was one of the five lncRNAs transcribed from *CDKN1A*. PANDA antisense transcription was found to be exclusively induced by p53 and this lncRNA sequesters the transcription factor NF-YA, which binds the promoter of apoptotic activators such as APAF1, BIK, FAS and LRDD (Figure 2C) [153]. Hence, PANDA counteracts CDKN1 inhibitory effect on the cell cycle and this may have implications in cancerous cell growth. The importance of untangling the networking mechanisms by which lncRNAs act as ceRNAs for miRNAs, proteins and DNA in cancer will provide novel and more precise therapeutic targets for cancer therapy.

#### 4.1.2. Pseudogenes Derived-lncRNAs

Pseudogenes represent duplicative, homologous sequence regions of their parental genes. For a long time, they were considered “junk DNA” non-coding genes defined also as a relic of evolution, without any function. Since the discovery in 1977 of the first pseudogene, identified in the 3′-end of its parental gene, 5S DNA of *Xenopus laevis*, the field of pseudogenes is finally starting to be important in malignancy pathways [154]. Pseudogenes may also have their origin through a retrotransposition of processed mRNAs back into the genome. There are different types of pseudogenes depending on their origin in the genome and are reviewed elsewhere [155]. Here we will highlight some examples of pseudogenes derived-lncRNAs in cancer. Pseudogenes act like “endogenous competitors” and affect miRNA binding on all their gene targets. Poliseno and co-workers identified the first pseudogene derived-lncRNA in an oncogenic networking, the tumor-suppressor gene phosphatase and tensin homolog pseudogene1 (PTENpg1) that sequesters numerous PTEN-targeting miRNAs by acting as a miRNA sponge [156]. They found that expression of PTENpg1 and PTEN was higher in normal human tissues compared to prostate tumor samples. Later, another study revealed that PTENpg1 transcribes two anti-sense RNA isoforms -α and -β (PTENpg1 asRNA-α, and -β) and that they function in an opposite way. The PTENpg1 asRNA α negatively regulates PTEN expression and the asRNA β is required for PTENpg1 (sense) to act as a miRNA decoy [157]. Depletion of PTENpg1 asRNA α in human osteosarcoma cell lines arrested their growth and sensitized cells to the DNA-damaging agent doxorubicin. The knowledge of this PTENpg1 and other pseudogene ncRNA regulatory pathways may prove therapeutically relevant in order to control gene expression and simultaneously provide valuable general information of tumor biology. Another pseudogene derived-lncRNA is the small ubiquitin-like modifier 1 pseudogene 3, (SUMO1P3). It was found to be upregulated in human gastric cancer tissues compared with paired-adjacent non-tumorous tissues thus, SUMO1P3 could be useful as a biomarker and a therapeutic target for GC [144]. Additionally, the Double Homeobox A Pseudogene 8 (*DUXAP8*) expressed-lncRNA, enhances gastric cancer cell proliferation and tumorigenesis through silencing PLEKHO1 transcription epigenetically, by binding to EZH2 and SUZ12 [158]. The same pseudogene was highly expressed in colorectal cancer (CRC) and its expression was positively associated with tumor size, pathological stage and lymphatic metastasis and was exerting its oncogenic function through epigenetically silencing the tumor suppressors *p21* and *PTEN* expression [159]. Such observations are critical to our current understanding of gene regulation and highlight a biological role for pseudogene-expressed lncRNAs in human cells.

#### 4.1.3. Decoying Role of circRNAs

A combination of computational, functional and biochemical analyses revealed that the antisense Cerebellar Degeneration-Related protein 1 (CDR1as), a known human circRNA, is conserved from annelids to humans, and contains 74 miR-7 seed matches [108,160]. Additionally, miR-671 cleaves CDR1as in an Ago2-slicer-dependent manner reducing CDR1 mRNA levels [161]. These studies reveal the sponging-sequestering effect of circRNAs on miRNAs. The biological significance of circRNAs decoying effect on miRNAs in cancer was further strengthened by an interesting study which unraveled that ciRS-7 (CDR1as) sequesters the tumor suppressor miR-7. This miRNA, suppresses EGF receptor in glioblastoma, IRS-1 and IRS-2, Raf1 and other oncogenes [162]. The ciRS-7/miR-7/miR-671 axis is quite intriguing and gives new insights of circRNA and miRNAs interaction in cancer. CiRS-7 inhibits miR-7 repression of its target genes and the miR-671 may liberate miR-7, upon cleavage of ciRS-7 (Figure 2D).

Recently, a small number of circRNAs, which sequester miRNAs have been reported but their functional role needs further investigation. The SRY circRNA was discovered to harbor complementary sequences for miRNAs and contains 16 binding sites for miR-138 [17,160]. More recently, another study identified circRNA-ZNF91 derived from a gene encoding a primate-specific zing-finger protein, ZNF91, with 39 additional sites for miR-296. CDR1as has 22 binding sites for miR-876-5p/3167 family [163]. In addition, the circular isoform of the coding gene Itchy E3 Ubiquitin Protein Ligase (ITCH), cir-ITCH functions as a sponge for miR-7, miR-17, and miR-214 in esophageal squamous cell carcinoma (ESCC). The same miRNAs bind to the 3′UTR of mRNA ITCH. Consequently, cir-ITCH increases the level of ITCH mRNA and promotes ubiquitination and degradation of phosphorylated Dvl2, thereby inhibiting the Wnt/β-catenin pathway and this reveals the anti-tumoral property of cir-ITCH [164]. Furthermore, decreased cir-ITCH level was associated with poor survival of glioma patients, by sponging miR-214 and regulating ITCH-Wnt/β-catenin pathway [165]. Therefore, restoration of cir-ITCH expression could be a future direction to develop a novel treatment strategy in ESCC and glioma. CircRNAs can also interact with proteins and an example comes from a study in which the circ-forkhead box O3 (circ-Foxo3) forms a ternary complex with two cell cycle regulatory proteins, p21 and CDK2 and blocks the cell cycle progression in NIH3T3 cells (Figure 2E) [166]. An analysis of Foxo3 gene revealed that a pseudogene with high homology to Foxo3 mRNA as well as a circ-Foxo3, was generated from the same *Foxo3* gene [167]. *Foxo3* pseudogene (*Foxo3P*), mRNA and circ-Foxo3 had the same potential binding sites for 8 miRNAs (three of them are miR-22, miR-138, miR-433) and they could sponge these miRNAs and increase the tumor suppressor gene Foxo3 translation [168]. Over-expression of Foxo3P, Foxo3 and circ-Foxo3 in a breast cancer cell line decreased cell proliferation and induced extensive cell death due to apoptosis in tumors formed by the same cell line in nude mice. Among the aforementioned Foxo3 molecules, circ-Foxo3 demonstrated to have a more potent miRNA sponging effect and on cell survival. This may be due to the stability of the circ- Foxo3. As previously mentioned, circRNAs have a longer half-life than the linear mRNAs and are also resistant to RNA exonucleases. Nevertheless, the intense investigations on function of circRNAs as a miRNA trap are hampered, mainly because of the missing loss-of function experiments due to an overlapping of the linear and circular isoforms. It has been also speculated that the small “free” circRNAs could be carried by exosomes outside of the cells and this could explain the low abundance of circRNAs [169]. There is ample evidence that the quantity of circRNAs may exceed their linear isoforms, depending of the cell type and tissue and may play important role either in progression of cancer or tumor suppression. This may have therapeutic implications for some types of cancer and although this field is still in its infancy, a future circRNA-based cancer therapy could become realistic.

#### 4.1.4. Progenitor miRNA

A novel paradigm of a miRNA post-transcriptional mechanism is the discovery of an intermediate progenitor miRNA described in the biogenesis of the polycistronic onco-miR-17-92 [170]. During the Emryonic Stem Cell (ESC) differentiation, Gregory and his colleagues noticed that there is an accumulation of the miR-17-92 cluster, but the levels of miR-92a, one of the members of the cluster, were stable. They discovered that the reason behind this accumulation was the presence of an inhibitory domain at the 5′ part of the pri-miR-17-92 that brought it to a higher-order RNA conformation. Therefore, the microprocessor complex was unable to process the primary transcript into the precursors. When the 5′ inhibitory domain was cleaved at 9nt before pri-miR-17a loop by the endonuclease CPSF3 aided by the spliceosome-associated ISY1, enabled the formation of a novel intermediate structure, named the progenitor miR-17-18a-19a-20a-19a except miR-92a. The latter was processed by Drosha, creating the 3′-end of pro-miR-17-19a. Importantly, the pro-miR formation was a necessary intermediate step for the miR-17-92 maturation. The same group proposed the biological relevance of this additional post-transcriptional step that could explain the opposite anti-tumoral effect of one of the members of this family, miR-92a, observed in mouse lymphoma [171]. Expression of the miR-17-92 cluster is increased in B cell lymphoma, T cell acute lymphoblastic leukemia (T-ALL) and other tumors. Interestingly, miR-92a inhibits oncogenic miR-19, which belongs to the same family, and promotes proteosomal degradation of c-Myc. In contrast, when miR-19 is more expressed, it overcomes the inhibitory action of miR-92 and promotes tumor progression. Overall, the processing of the cluster pri-miR-17-92 by CPSF3 and ISY1, gives rise to a pro-miRNA that omits miR-92a from the cluster. This could explain the antagonism between miRNAs belonging in the same cluster with a high impact on tumor formation. 

## 5. Disruption of ncRNA Networks in Cancer

### 5.1. miRNA Editing

**Figure 3:**
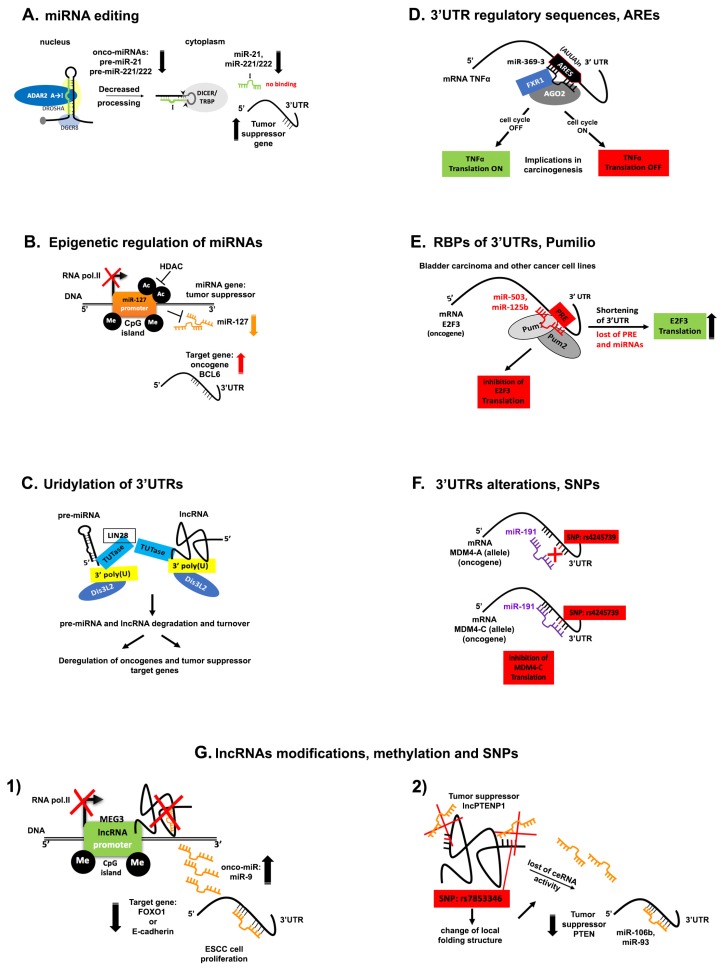
Disruption of ncRNA Networks in Cancer. (**A**): miRNA editing: in glioblastoma, ADAR2 edit the precursors of miR-222/221 and miR-21 and decrease expression of the corresponding oncomiRs in vitro and in vivo, (**B)**: Epigenetic regulation: Methylation and deacetylation of miR-127 promoter suppresses expression of the tumor suppressor miRNA. Consequently, BCL6 oncogene expression increases. (**C**): Uridylation of miRNA and lncRNA: the enzyme TUTase adds Uridine groups at the 3′-end of pre-*let-7* and the lncRNA Rmrp. Degradation of oligo-Uridine tails through the exonuclease Dis3L2 unified in the DMD pathway may explain the reduced expression of *let-7* and other tumor suppressor miRNAs as well as lncRNAs deregulation in cancer. (**D**): 3′UTR regulatory sequences, ARES: ARES interact with micro-ribonucleoproteins (microRNPs) such as AGO2 as a complex with other proteins to regulate the stability and translation of mRNAs in response to external or internal stimuli. miR-369-3 binds ARES, AGO2 and fragile X mental retardation-related protein 1 (FXR1) complex to induce translation of TNFα, in serum starved HEK293 and HeLa cell lines. When cells start to proliferate, miR-369-3 inhibits TNFα. (**E**): RBPs of 3′UTR, Pumilio: Bladder carcinomas selectively down-regulate miRNAs that cooperate with Pumilio to target E2F3, and multiple tumor cell lines shorten the 3′-end of the E2F3 mRNA, removing the Pumilio regulatory elements. (**F**): 3′UTR alterations SNPs: A somatic SNP in the 3′UTR of the oncogene *MDM4* created a new binding site for miR-191. This miRNA suppressed only the oncogene *MDM4-C* allelic variant expression and retarded ovarian cancer progression and the tumor became chemosensitive. (**G**): LncRNA modifications: (**1**) Methylation: tumor suppressor, lncRNA MEG3, has binding sites for onco-miR-9, so that it does not bind its targets, FOXO1 and E-cadherin, in ESCC human cell lines. Promoter of lncRNA MEG3 is usually methylated in ESCC and cannot sequester miR-9 and this induces to proliferation of cancer cells. (**2**) SNPs: lncRNA PTENP1 polymorphism rs7853346 in GC, change the local folding structure of lncRNAPTENP1. The latter is not able to exert its decoying effect on miR-106b and miR-93, with a consequent reduction of the tumor suppressor *PTEN* gene expression.

Adenosine to inosine (A-to-I) editing catalyzed by adenosine deaminases acting on RNAs (ADARs) affects miRNA biogenesis and reprograms miRNA targeting [172]. Thousands of hitherto unknown A-to-I RNA editing sites in human transcripts, in normal tissues and in various cancers have been identified thanks to the advanced deep sequencing technology and bioinformatics analysis [173]. Editing sites are typically found in introns and in 3′UTRs where inverted Alu repeats form double stranded RNAs (dsRNAs) [174,175,176]. Therefore, Alu elements are the substrates of RNA editing. A-to-I RNA editing by ADAR of pri- and pre-miRNAs may regulate expression and function of their mature counterparts. ADAR enzymes form a heterodimer complex with Dicer to promote miRNA processing or inhibit the microprocessor complex cleavage of pri-miRNAs and therefore suppress miRNA maturation [172,177]. In turn, these enzymes are regulated by CREB and c-Jun transcription factors. In glioblastomas, editing within miRNA is decreased or lost because the activity of ADAR2 is impaired compared with the normal brain tissue [178]. Upon reconstitution of ADAR2 activity, miRNA editing was recovered and the expression of onco-miRs and onco-suppressors miRNAs became balanced as seen in the normal brain tissue. Surprisingly, ADAR2 edited the precursors of miR-222/221 and miR-21 and decreased the expression of the corresponding oncomiRs in vitro and in vivo (Figure 3A). In another study, in situ analysis of melanoma samples in microarrays tissue was performed [179]. This study reported that expression of ADAR1 was frequently reduced during the metastatic transition. Moreover, knockdown of ADAR1 changed the cell phenotype of 131 cancer regulators miRNAs and microarray analysis revealed that ADAR1 controls the expression of miRNAs that target genes associated with these phenotypic changes. Furthermore, overexpression of the onco-miR, miR-17-5p and a newly identified miR-432 silenced both isoforms of ADAR1 in melanoma cells. A recent study in melanoma identified that miR-455-5p has two A-to-I editing sites in low metastatic melanomas and this changed its inhibitory effect on its target, the tumor suppressor gene, *CPEB1* [180]. In other words, A-to-I editing of miR-455-5p changed completely its biological effect and inhibited melanoma growth and metastasis. In addition, a study revealed that overexpression of ADAR1 in chordoma, a rare neoplasm of the axial skeleton, accompanied reduced levels of miR-10a and miR-125a, caused by enhanced pre-miR-10a and pri-miR-125a A-to-I editing [181]. This phenomenon might contribute to the pathogenesis of chordoma.

In summary, ADAR enzymes seem to have both anti-tumoral or a pro-tumoral effect in different types of cancer by reducing the expression of onco-miRs or tumor suppressors miRs. These enzymes thus represent promising targets for tumor therapy.

### 5.2. Epigenetic Regulation of miRNAs

It is estimated that 40% of the human genes promoter contain Cytosine-phosphodiester bond-Guanosine (CpG) islands, at the 5′ end of regulatory genes [182]. Many studies have reported that the 5-carbon of cytosine pyrimidine ring can be methylated and methylation of tumor suppressor genes may lead to cancer. Since many miRNA promoters are embedded in (CpG) islands of their host genes involved in neoplastic development, it is reasonable that methylation of these DNA regions of tumor suppressive miRNAs may lead to the development and progression of cancers. Furthermore, another epigenetic modification of miRNA promoters is the histone H3 and H4 hypo-acetylation by Histone Deacetylation (HDAC) enzymes [183]. Evidence that miRNA expression is regulated through epigenetic modifications of their promoter came from studies of cancerous and healthy cells treated with DNA-demethylating agents, like 5-Aza-2′-deoxycytidine (5-Aza-CdR) and histone deacetylase inhibitor, 4-phenylbutyric acid (PBA). It has been reported that the promoter of miR-127 is inserted in CpG islands and it is silenced in primary tumors of prostate, bladder, and colon [184]. After a combinatorial treatment of these cell lines with 5-Aza-CdR and PBA, miR-127 expression was restored and its target, the proto-oncogene B-cell lymphoma-6 (*BCL-6*) was repressed (Figure 3B) [185]. Chromatin immunoprecipitation (ChIP) showed that activation of miR-127 expression after treatment was due to decreased cytosine methylation, H3 acetylation and methylation of H3 at lysine 4 (H3-K4), around the transcriptional start sites of this gene. Another study has examined the miRNA methylation profile of human metastatic cancer cells of colon, melanoma and head and neck cancer [186]. Among other miRNAs, it was found that the CpG islands in the promoter of miR-34b/c, miR-148 and miR-9 were hypermethylated, therefore underexpressed and their target genes expression, *c-myc* and *CDK6* were consequently highly expressed. Surprisingly, it was observed that hypermethylation of these miRNAs was associated with the metastatic cancer cells in the corresponding lymph nodes. This finding highlights a promising application of the miRNA methylation profile as a metastatic marker. Recently, a high-throughput sequencing analysis investigated how chemoresistance of a breast cancer cell line to adriamycin (ADM) and paclitaxel (PTX) can influence gene expression, methylation status and miRNA expression in comparison to chemosensitive controls [187]. This study reported that highly expressed miRNAs in the chemoresistant cells were less methylated around their transcriptional start site (TSS) and vice versa. Furthermore, investigators have analyzed genes with Kyoto Encyclopedia Of Genes and Genomes (KEGG) database, that were regulated by methylation and miRNAs. Seventeen genes were identified to be associated with methylation and dysregulation of miRNAs and generally they were involved in the cell motility and EMT pathways. Overall, these studies indicate that epigenetic mechanisms may control transcription of tumor suppressor miRNAs. Methylation profile of miRNAs can be engaged as a prognostic marker as well as for metastatic propensity. Moreover, treatment of tumors with drugs that interrupt this control may have therapeutic implications for cancer cure. 

### 5.3. Uridylation of the 3′-End of ncRNAs

Several studies in plants and animal models indicate that uridylation regulates ncRNAs whereas longer mRNAs are degraded after addition of uridines [188,189]. The only known substrate in the nucleus of uridylation is the small nucleolar (snοRNA) RNA U6, operated by the U6 Terminal Uridylyl Transferase (TUTase), an essential enzyme for cell survival in mammals [190]. The addition of four Uridine residues in the 3′-end of U6 is essential for its splicing function. In the cytoplasm, polyuridylation occurs on polyadenylated and non-polyadenylated RNA such as miRNAs and has been shown to regulate miRNA biogenesis and activity [191].

Deep sequencing analysis with strand specific RNA linker ligation to identify and examine the 3′-ends of small RNAs in human embryonic stem cells, suggested that the largest non-template modification was uridylation, especially in mature miRNAs, such as miR-302-367 cluster [192]. A further high-throughput sequencing analysis specifically for miRNA precursors was performed to better understand the mechanism and function of oligo-uridylation in the 3′-end of miRNAs [193]. Many pre-microRNAs were found to be uridylated and this modification was increased in differentiated adult mouse tissues. Among pre-miRNAs, pre-*let-7* family was identified. It is known from previous studies that *let-7* precursor is uridylated at the 3′-end by the uridylyl transferase, TUTase4 (TUT4) (Figure 3C) [194]. In this mechanism, Lin28 binds the conserved sequence motif GGAG in the terminal loop of pre-*let-7* and triggers the Terminal Uridylyl Transferases 4 and 7 (TUT4/7). This finally inhibits *let-7* maturation in embryonic stem cells affecting their maintenance. Dis3L2, an RNase II/3′-5′exonuclease, which is the catalytic subunit of the RNA exosome recognizes and degrades the oligo-uridylated pre-*let-7* [195,196]. This enzyme is mutated in the Perlman syndrome and in Wilms tumors and its role in tumorigenesis is only recently unraveled after the discovery of the Dis3L2-Mediated Decay (DMD) pathway [197,198]. An analysis of Dis3L2 targets in mouse embryonic stem cells after an RNA immunoprecipitation assay identified lncRNAs and pseudogenes in DMD pathway. A series of in vitro Dis3L2 loss-of- function experiments helped to determine that some of the lncRNAs were 3′ uridylated and as expected they were accumulated in the cytoplasm (Figure 3C). Deep sequencing of the 3′-ends particularly of the RNA Component of Mitochondrial RNA Processing Endoribonuclease lncRNA (Rmrp), identified that the uridine tail was added to those which contained CAC nucleotides after the 3′-end and thereby were degraded by Dis3L2. DMD pathway is a guardian sentinel of the aberrant oligo-uridylated RNA species that should be degraded especially when these RNAs are cancer-related. The ncRNAs and the subsequent degradation of oligo-Uridine tails through Dis3L2 enzyme unified in the DMD pathway may explain the reduced expression of *let-7* and other tumor suppressor miRNAs as well as lncRNAs deregulated in cancer [199]. 

### 5.4. Phosphorylation of the 5′ End of miRNAs 

The mere presence and detection of a miRNA in the cytoplasm does not imply its activity. Indeed, a miRNA is active when it is able to repress its target genes unless its activity is compromised by various alterations, as described earlier. 

A novel paradigm of a miRNA modification that enhances its activation has been recently discovered and it is the phosphorylation of the 5′ end in the mature tumor suppressor miR-34 [200]. This miRNA is usually methylated thereby under-expressed in human malignancies or because miR-34 gene locus, 1p36, is frequently deleted in human tumors [201]. Although in some instances, it can also be present abundantly in human cancer cell lines [202]. It is also known that p53 binds to the promoter of miR-34 and enhances the transcription thereof, especially when a DNA damage external signal is triggered. Furthermore, many miRNAs when knocked down, do not influence important biological functions but might respond to external stimulus, such as irradiation. In fact, in this study a pool of mature and inactive miR-34 was detected in cancer cell lines. But when the same cells underwent ionizing irradiation (IR) to induce DNA damage, this pool became activated. The activation was through the human RNA 5′-kinase (hClp1) and Ataxia telangiectasia (ATM)-dependent 5′ phosphorylation of the mature miR-34. Interestingly, 5′ phosphorylated miR-34a suppressed its targets genes, Cyclin Dependent Kinase 4 (*CDK4*) and B-cell Lymphoma 2 (*BCL2*). This surprising finding may explain why regardless of a high expression of miRNAs, sometimes they might not be functionally active. More importantly, this innovative modification could be further explored in other tumor suppressor miRNAs as a rapid cellular defense to external stimuli. This study provides an interesting perspective for a cancer therapy based on the activation of the existing inactive tumor suppressor pool inside the cytoplasm rather than injecting miRNAs intravenously in human patients.

### 5.5. Alterations and Regulatory Sequences within 3′UTRs

The 3′ untranslated regions (3′UTRs) of mRNAs is defined as the sequence between the STOP codon and the poly(A) tail. It is important for their transportation into the cytoplasm, the stability and translational efficiency of mRNAs. These regions are targeted by miRNAs, therefore length variations of 3′UTRs of many genes affect their regulation by miRNAs. These alterations derive from the alternative polyadenylation mechanism (APA) present in more than 70% of the mammalian genes. This leads to formation of isoforms with multiple 3′UTRs derived from a single gene. The length of a 3′UTR is dictated by alternative splicing and more frequently by the cleavage and polyadenylation of the pre-mRNA at defined sequences, 10–30 nt after the canonical sequence AAUAAA or its variants known as polyadenylation sites (PAS) [203,204]. Moreover, short 3′UTRs are more stable therefore more abundant because they are no longer recognized by miRNAs to suppress the translation. This phenomenon is frequently observed in actively proliferating cells and in cancer [205]. 

Equally important and well connected to APA mechanism is the presence of regulatory sequences within 3′UTRs. An example is the ubiquity of Adenosine-Uracil elements (ARES), which are conserved sequences within mRNA 3′UTRs. ARES interact with micro-ribonucleoproteins (microRNPs) such as AGO2 in complex with other proteins to regulate the stability and translation of mRNAs in response to external or internal stimuli [206]. A seminal observation that miRNAs may induce mRNA translation demonstrated that miR-369-3, interact with AGO2 and fragile X mental retardation-related protein 1 (FXR1) complex to induce translation of TNFα, in serum starved HEK293 and HeLa cell lines (Figure 3D). Therefore, upon cell cycle arrest, the interaction between the seed sequence of the miRNA within the minimal TNFα ARE 3′UTR induces TNFα translation but in proliferating cells, miRNA had exactly the opposite inhibitory effect [207]. Similarly, *let-7* enhanced translation of its target mRNA in quiescent cells and repressed it when the cells were proliferating. 

In another study, a translational control element (TCE) within the Kruppel Like Factor (KLF4) 3′UTR interacted with miR-206 and miR-344-1 to promote or inhibit KLF4 expression respectively, in proliferating normal human mammary epithelial cells [208]. miR-206 induced translation of KLF4 to enhance the malignant phenotype in human mammary epithelial cells. 

Overall, trans-factors such as RNA binding proteins (RBP) and miRNAs act to contend or cooperate with each other in order to regulate cis-regulatory sequences within mRNAs involved in cancer [209,210,211]. Another example of this mechanism includes the RBP Human antigen R (HuR), a member of the human embryonic lethal abnormal visual (Elav) gene family that stabilizes and regulates translation of mRNA [212,213]. Recently, a dissecting study of the *KRAS* 3′UTR oncogene in HeLa cells identified two 49-nt cis-regulatory sequences and miR-185 was able to bind one, while the other contained a stabilizing element [210]. Furthermore, knocking down of HuR and Dicer has increased mRNA and protein levels of KRAS. These results indicate that miRNA and HuR cooperatively bind the same sequence in the 3′UTR of this oncogene and repress its expression. Moreover, in the same repressive 49-nt sequence there was a single nucleotide polymorphism (SNP) at the first nucleotide of the predicted miR-185 target site but its role in the tumorigenesis has yet to be determined in the above context. 

Other RBP reported to be involved in human malignancies, is the human Pumilio protein complex, which upon binding to cis-regulatory sequences within 3′UTRs, induces a conformational change in the RNA structure and regulates miRNAs activity towards their target genes [214,215,216]. Bladder carcinomas selectively down-regulate miR-503 and miR-125b that cooperate with Pumilio to target E2F3, and multiple tumor cell lines shorten the 3′-end of the E2F3 mRNA, removing the Pumilio regulatory elements (Figure 3E) [217]. These observations indicate one of the ways how oncogenes escape the inhibitory effect of tumor suppressor miRNAs. Among the RBPs that regulate miRNA targeting on mRNAs in the nucleoplasm and cytoplasm, the Splicing factor proline/glutamine-rich protein (Sfpq) promotes *let-7a*-mediated degradation on *Lin28* 3′UTR mRNA through a nuclear mechanism in human and mice cell lines [54]. The multitude of actions of many other RBP proteins known to interfere with binding sites within 3′UTRs of genes involved in tumorigenesis and influence miRNAs seed sequences still need to be explored [218]. Altogether, interactive networks between 3′UTRs, RBP, miRNAs and specific cellular conditions entail a new window of opportunity for cancer therapy, by repressing or enhancing important interactions within these networks.

As mentioned above, in the general view of 3′UTR aberrations are the single nucleotide polymorphism (SNPs) that interrupt cis-acting regulatory elements embedded in the 3′UTR as well as chromosomal translocations that involve the 3′UTR resulting in the loss of miRNA complementary sites [219].

The SNPs are involved in many types of cancers and they occur frequently in the human genome, at about every hundred base pairs (bp) [220]. Interestingly, SNPs are usually silent mutations of a single nucleotide, which do not alter the amino acid sequence, however, there are nonsynonymous SNPs that change the amino acid sequence to create a new protein and gene function. The silent polymorphisms may reside in non-coding genomic regions like miRNAs and 3′UTRs. When polymorphisms fall into an important region of the miRNA seed sequence the regulation of its target genes will be abrogated. Since the first study of a wide-genome analysis DNA sequencing of tumor tissues whereby 12 miRNA-binding SNPs were identified to be associated with human cancer, many more studies have emerged [221]. For example, in lung cancer, a germline SNP in the 3′UTR of *KRAS* proto-oncogene abrogated the binding site of let-7, thus increasing KRAS protein levels and promoted tumor progression [222]. Moreover, a somatic SNP was found in the 3′UTR of the oncogene Mouse Double Minute 4 (*MDM4*) known to decrease p53 tumor suppressor function, which created a new binding site for miR-191. This miRNA suppressed only the *MDM4-C* allelic variant expression and retarded ovarian cancer progression and the tumor became chemosensitive (Figure 3F) [223]. A wide-genome analysis for SNPs associated with breast cancer within 3′UTRs that modify miRNA binding sites identified two SNPs, one in the *BRCA1* 3′UTR that created a binding site for the miR-638 and decreased the levels of BRCA1 protein in breast cancer cells whereas the other was in the *TGFBR1* 3′UTR and created a binding site for miR-268 [224]. These findings show that SNPs may turn out to be valuable as cancer biomarkers which affect prognosis as well as response to therapy. 

### 5.6. Genetic Alterations 

Since tumorigenesis involves genomic alterations, such as chromosomal translocations that may influence or produce proto-oncogenes and onco-suppressors as well as “fusion genes”, a recent pioneering study investigated the impact these genomic alterations may have on non-coding RNAs and specifically on circRNAs [225]. One example of fusion genes is the breakpoint cluster region protein-Abelson murine leukemia viral oncogene (*BCR-ABL*) gene on chromosome 22 or Philadelphia chromosome, discovered in leukemia patients. The investigators showed that upon chromosomal translocation, the two genes bring complementary repetitive intronic sequences (Alu), which generally favors back-splicing events to generate aberrant circRNAs, called fusion (f)-circRNAs. They demonstrated that many recurrent chromosomal translocations, of *PML/RARα* observed in Acute Promyelocytic Leukemia (APL), *MLL/AF9* in Acute Myeloid Leukemia (AML), *EWSR1/FLI1* in Ewing Sarcoma and *EML4/ALK1* associated with lung cancer could form f-circRNAs [226,227]. Interestingly, the f-circM9 derived from *MLL/AF9* fusion and f-circPR (derived from *PML/RARα* fusion), enhance proliferation of leukemia cells in vitro (Figure 2F). But the f-circRNAs failed to induce leukemia in mice. When both the f-circRNA and the fusion protein (f-protein) were overexpressed in leukemia cells and introduced in mice, they enhanced leukemia progression and resistance to chemotherapy. 

The intriguing role of circRNAs in tumorigenesis has only started to be uncovered and the number of circRNAs involved in cancer is progressively increasing. Computational identification of these remarkable ncRNAs as well as an accurate estimation of their abundance and function in every type of cancer will aid potential therapeutic approaches based on them.

### 5.7. lncRNA Modifications in Cancer

Dysregulated lncRNAs due to methylation or mutation in single nucleotides may occur in cancer. For example, the reason behind the low expression of lncRNA LOC100130476 in the pathogenesis of esophageal squamous cell carcinoma (ESCC) has been investigated in 123 ESCC patients comparing with the normal tissues. Low expression was due to hypermethylation of the CpG sites in exon 1 closing to the transcription start site of LOC100130476 [228]. The ESCC patients with low expression or hypermethylation of this lncRNA had a poor prognosis. The aberrant hypermethylation within the three CpG islands regions around the transcription start site of another lncRNA CTC-276P9 was identified in esophageal cancer cells and ESCC tissues and was also related with poor survival of ESCC patients [229]. In another study, the promoter region of maternally expressed gene 3 (MEG3) lncRNA was methylated in ESCC. Interestingly, this lncRNA has ceRNA functions. It sequesters the onco-miR-9 so that it does not target FOXO1 and E-cadherin in ESCC human cell lines, eventually leading to inhibition of tumor cell proliferation (Figure 3G1) [230]. The above examples identify three lncRNA as prognostic biomarkers and therapeutic targets for ESCC patients. Three lncRNA PTENP1 tagSNPs, namely, rs7853346 C>G, rs865005 C>T, and rs10971638 G>A were genotyped in 768 GC patients and 768 cancer-free controls in a Chinese population [231]. Those patients with rs7853346 G allele had significantly reduced risk of GC, compared with those carrying C allele and was more obvious in older subjects (≥60 years), nonsmokers, nondrinkers, and subjects without family history of GC. Through bioinformatics analyses, it was found that rs7853346 might change the local folding structure of lncPTENP1 abolishing its sponging effect on miR-106b and miR-93 with a consequent reduction of the tumor suppressor *PTEN* gene expression (Figure 3G2). These data suggest that GC susceptibility can be predicted by lncRNA PTENP1 polymorphism rs7853346 [231]. In another study it was found that the lncRNA H19 SNPs may contribute to susceptibility to GC resulting in gain and loss of function of miRNA-lncRNA interactions [232]. 

## 6. Perspectives

As the so-called dark matter ncRNAs gets more and more elucidated, we have started to uncover some fascinating concepts and paradigms showing how they interact with each other, both in normal and diseased circumstances. The role of miRNAs is definitely the most studied among ncRNAs, mainly because they have the advantage of having been discovered earlier than their longer kins. In order to broaden the knowledge of how lncRNAs contribute to cancer development, besides studying their networking ability to act as ceRNA for miRNA, proteins and DNA, the development of suitable animal model systems will be critical. Revelation of pseudogene-derived lncRNAs is an exciting new development and how these lncRNAs interact with miRNAs will provide novel biomarkers and prognostic tools for cancer. The relatively younger protagonists, namely circRNAs which act as miRNA traps and the fusion circRNAs generated during chromosomal translocation further emphasize how the complex network of ncRNAs is perturbed in cancer. A better individual and collective understanding of the members of this nefarious nexus will set the stage for RNA aided cancer therapy.
